# A GOOD END OF LIFE WITH DEMENTIA ACROSS CULTURES

**DOI:** 10.1093/geroni/igac059.939

**Published:** 2022-12-20

**Authors:** Jenny van der Steen

**Affiliations:** Leiden University Medical Center, Leiden, Zuid-Holland, Netherlands

## Abstract

Conceptualizations of a good end of life with dementia vary across cultures and understanding differences and similarities can help develop care planning tools and help understand validity of measuring it in the context of cross-cultural comparisons. We have analysed (2019-2020) 14 cross-cultural studies covering qualitative data of 121 people with dementia and 292 family caregivers. Nine cross-culturally common themes emerged, ranging from basic care needs addressed to satisfaction with life and spiritual well-being. Good relationships were essential in all themes, but an emphasis on “care for caregivers” differed the most across cultures. Based on this work, we expanded an existing dementia care goals model by refining palliative care goals beyond comfort and functioning, and in an ongoing Delphi study (2021-2022) it was evaluated by experts of over 30 countries. This session will report on these studies, focusing on relevant cross-cultural differences, and connecting it to available measures of comfort and end-of-life care.

